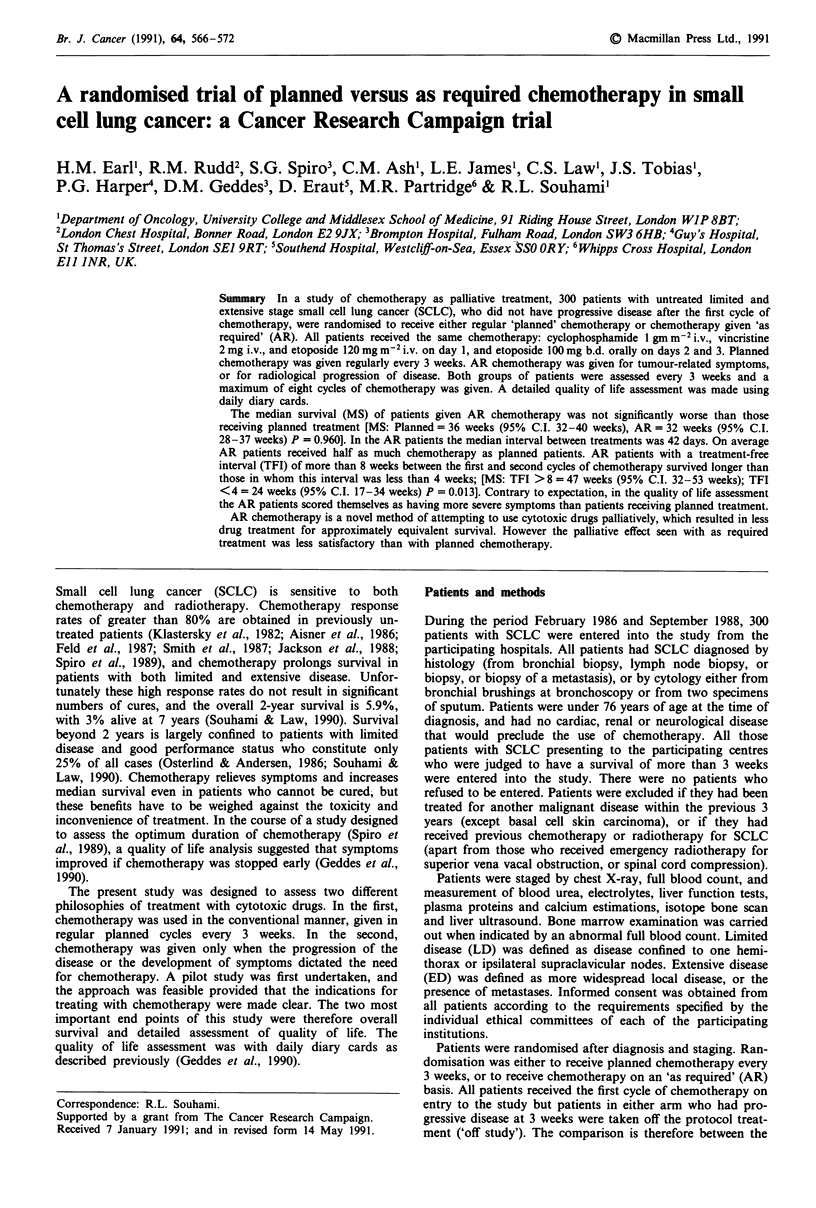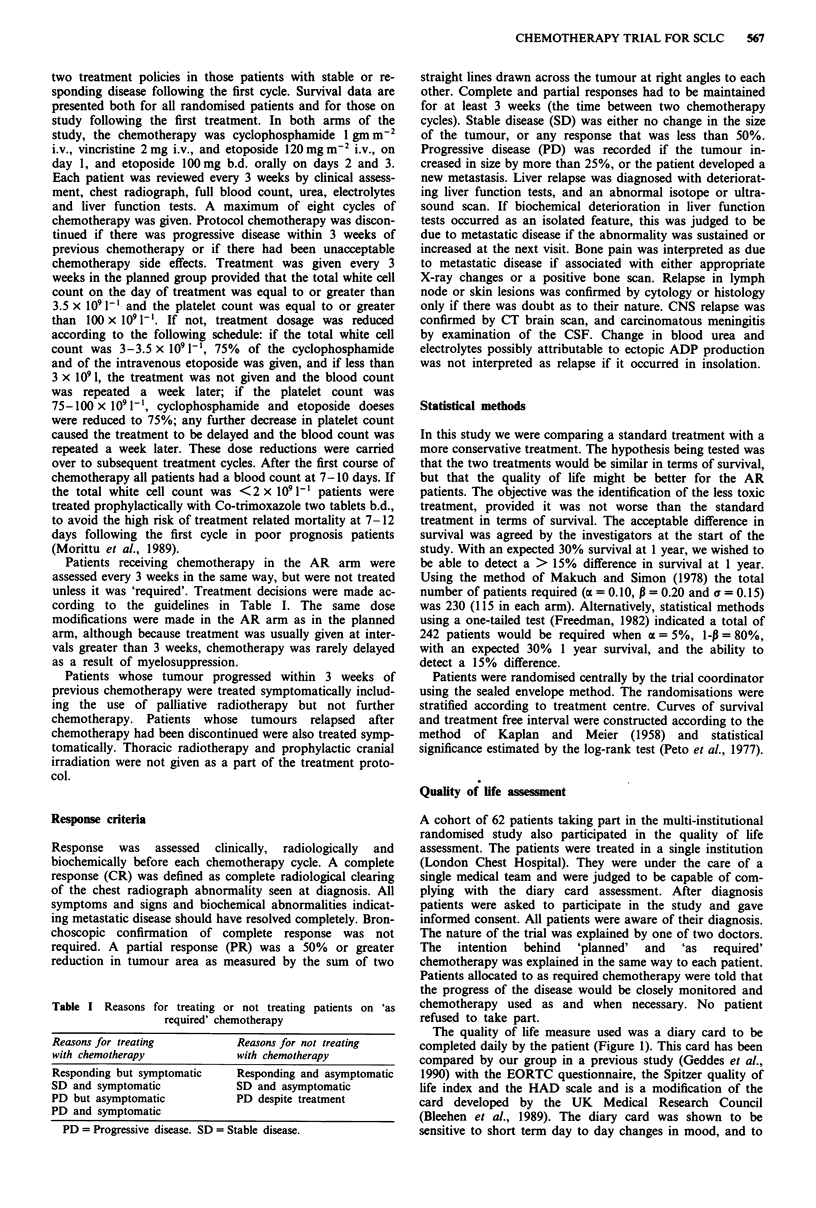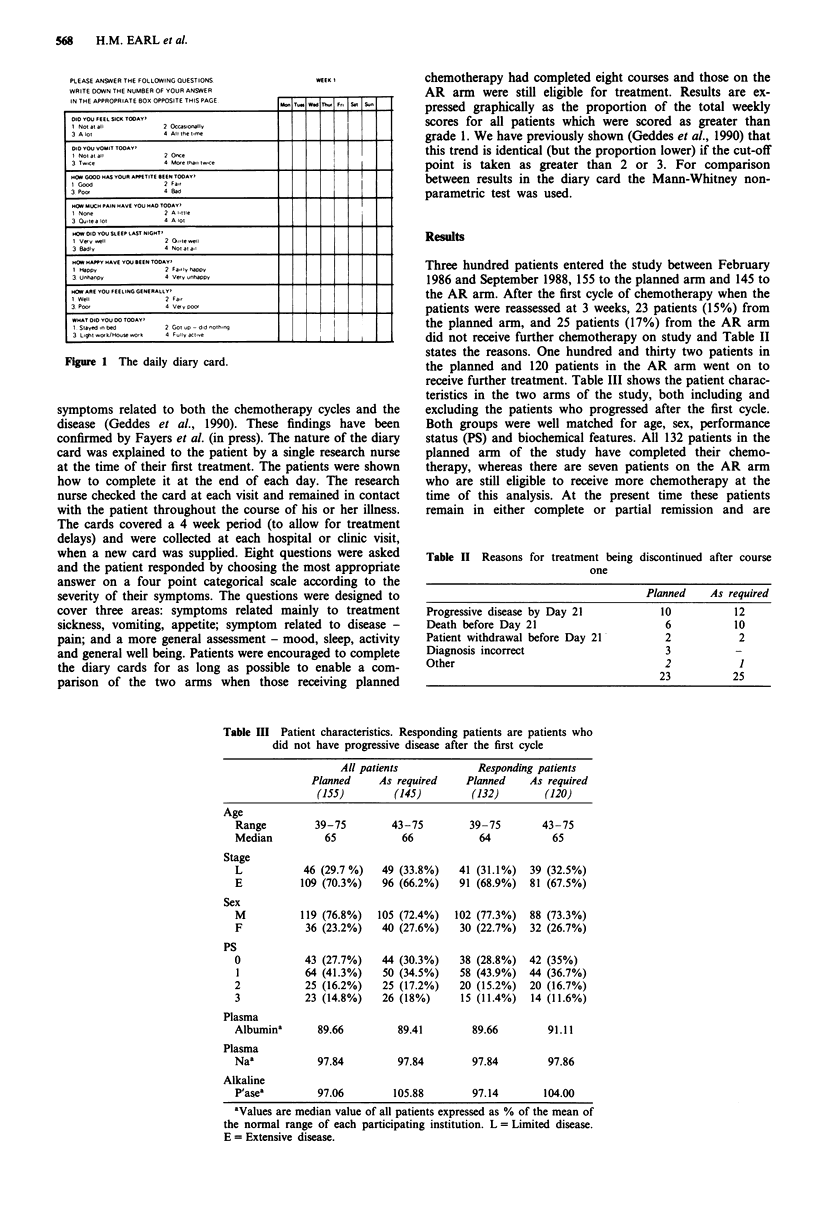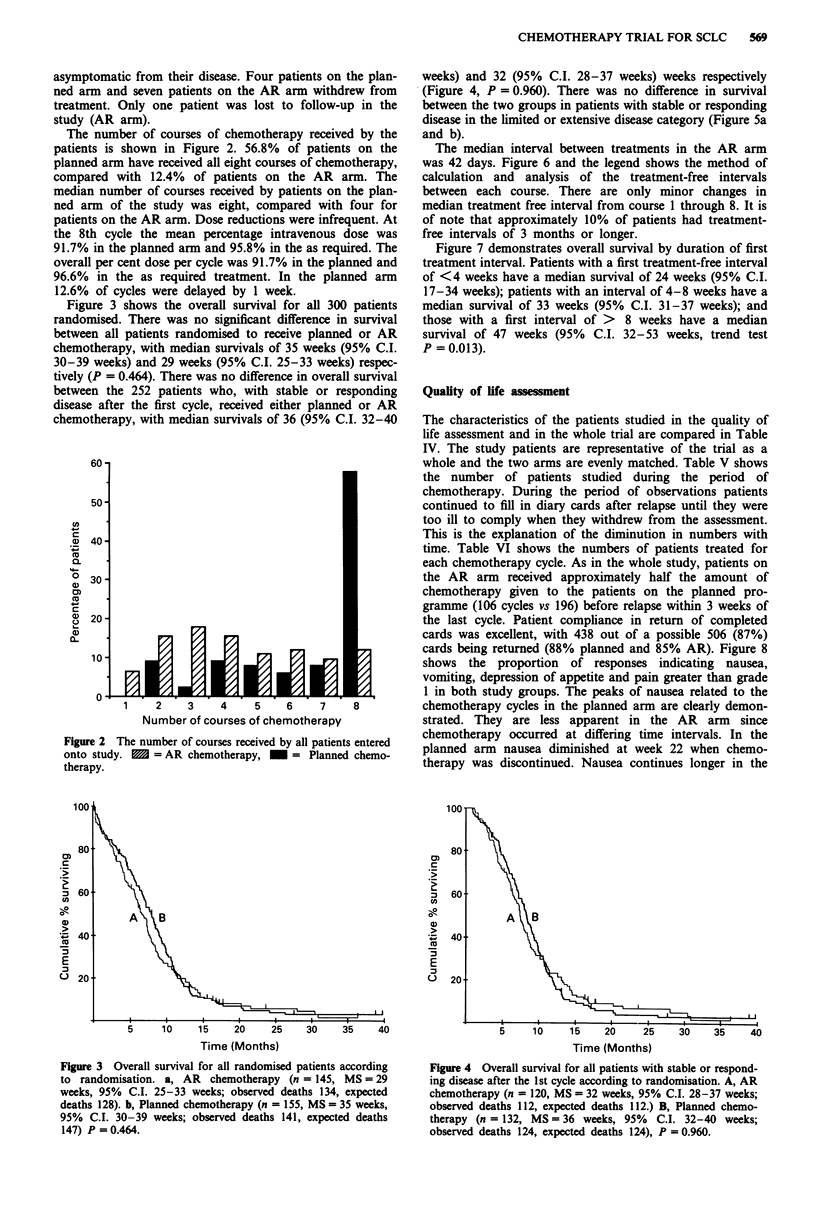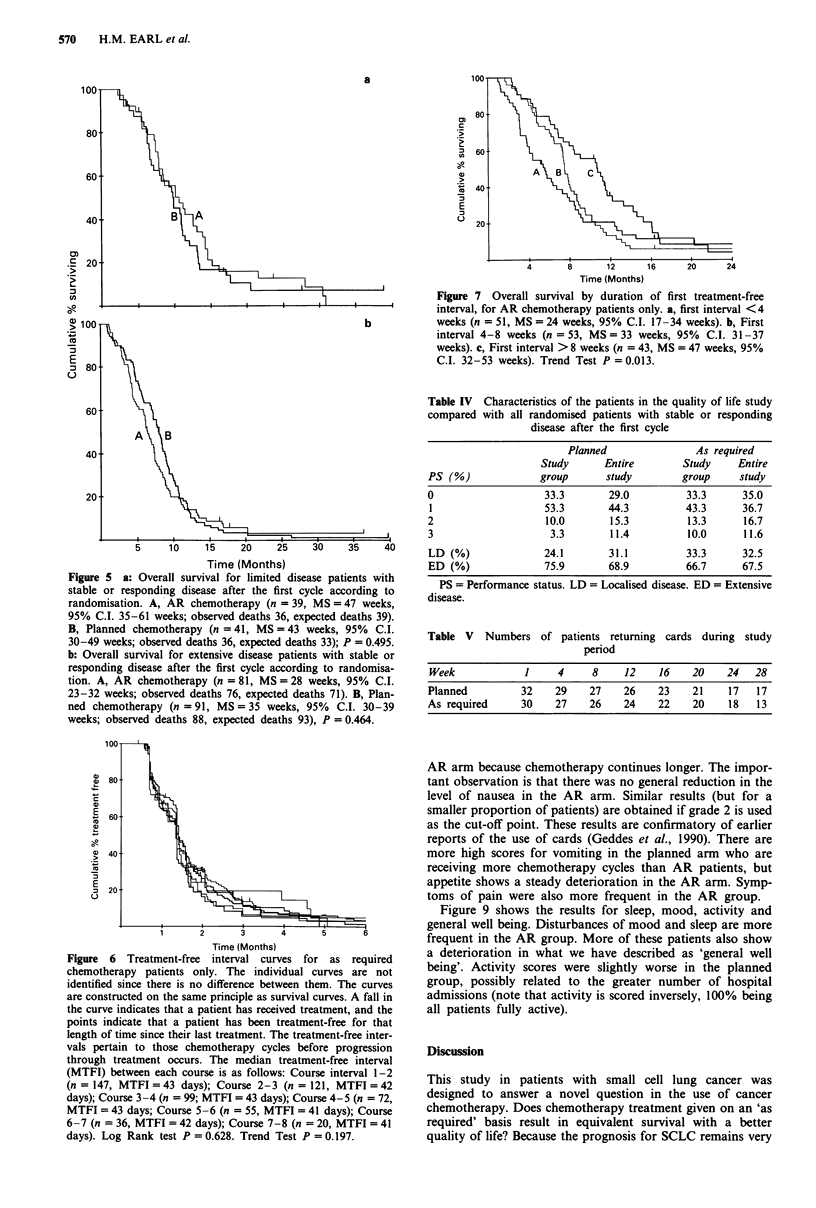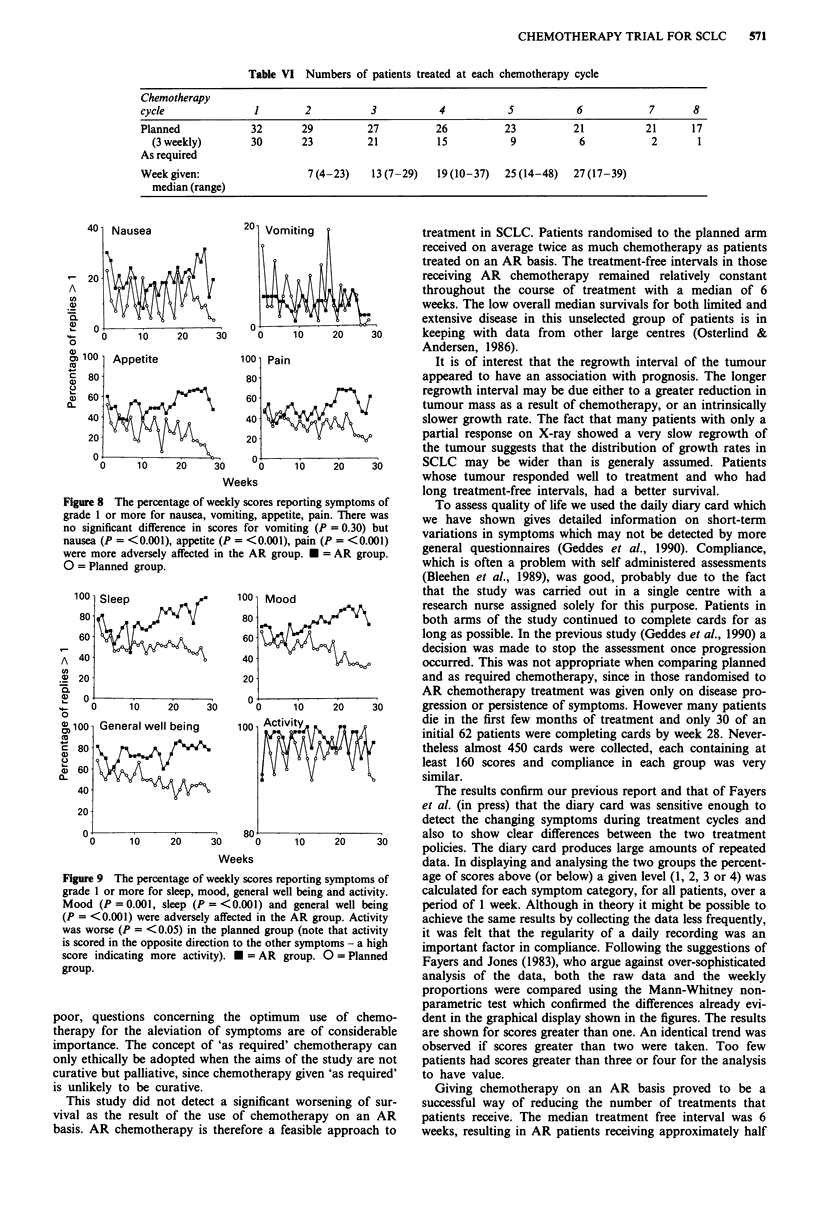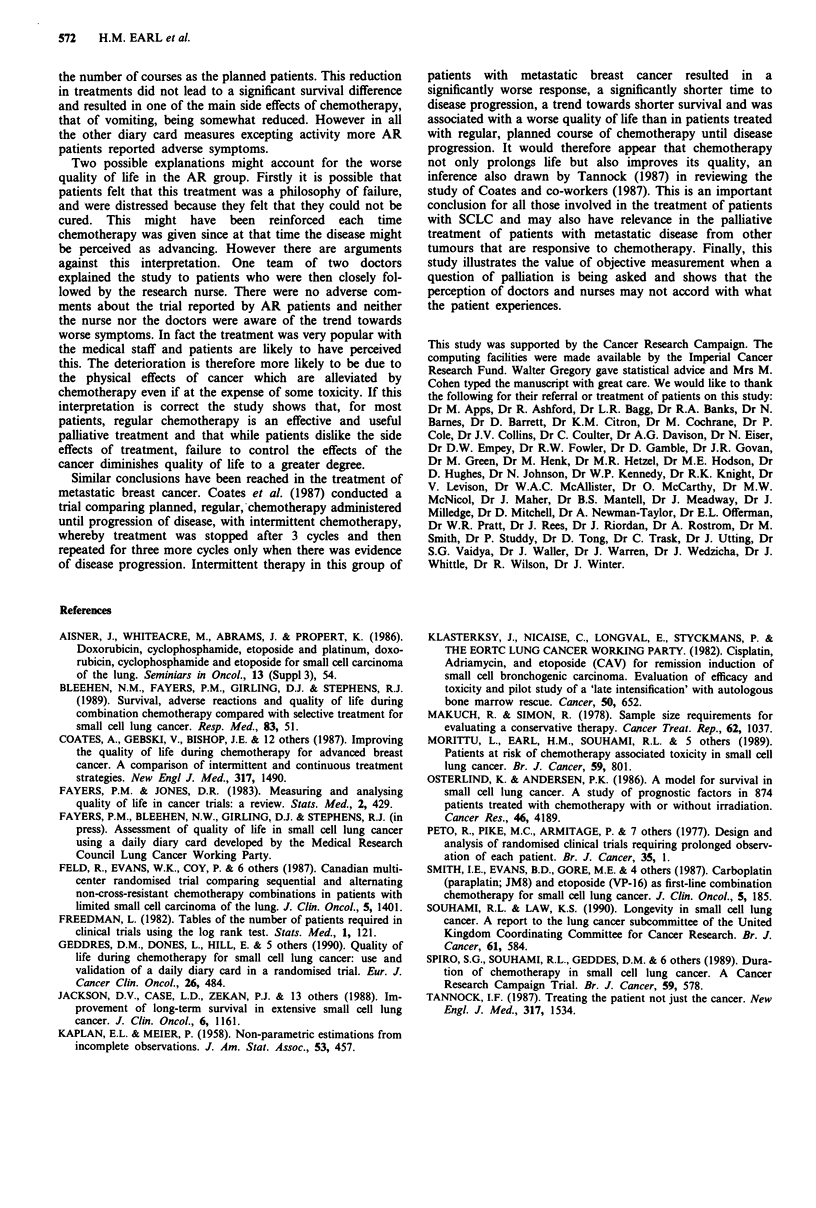# A randomised trial of planned versus as required chemotherapy in small cell lung cancer: a Cancer Research Campaign trial.

**DOI:** 10.1038/bjc.1991.351

**Published:** 1991-09

**Authors:** H. M. Earl, R. M. Rudd, S. G. Spiro, C. M. Ash, L. E. James, C. S. Law, J. S. Tobias, P. G. Harper, D. M. Geddes, D. Eraut

**Affiliations:** Department of Oncology, University College and Middlesex School of Medicine, London, UK.

## Abstract

In a study of chemotherapy as palliative treatment, 300 patients with untreated limited and extensive stage small cell lung cancer (SCLC), who did not have progressive disease after the first cycle of chemotherapy, were randomised to receive either regular 'planned' chemotherapy or chemotherapy given 'as required' (AR). All patients received the same chemotherapy: cyclophosphamide 1 gm m-2 i.v., vincristine 2 mg i.v., and etoposide 120 mg m-2 i.v. on day 1, and etoposide 100 mg b.d. orally on days 2 and 3. Planned chemotherapy was given regularly every 3 weeks. AR chemotherapy was given for tumour-related symptoms, or for radiological progression of disease. Both groups of patients were assessed every 3 weeks and a maximum of eight cycles of chemotherapy was given. A detailed quality of life assessment was made using daily diary cards. The median survival (MS) of patients given AR chemotherapy was not significantly worse than those receiving planned treatment [MS: Planned = 36 weeks (95% C.I. 32-40 weeks), AR = 32 weeks (95% C.I. 28-37 weeks) P = 0.960]. In the AR patients the median interval between treatments was 42 days. On average AR patients received half as much chemotherapy as planned patients. AR patients with a treatment-free interval (TFI) of more than 8 weeks between the first and second cycles of chemotherapy survived longer than those in whom this interval was less than 4 weeks; [MS: TFI greater than 8 = 47 weeks (95% C.I. 32-53 weeks); TFI less than 4 = 24 weeks (95% C.I. 17-34 weeks) P = 0.013]. Contrary to expectation, in the quality of life assessment the AR patients scored themselves as having more severe symptoms than patients receiving planned treatment. AR chemotherapy is a novel method of attempting to use cytotoxic drugs palliatively, which resulted in less drug treatment for approximately equivalent survival. However the palliative effect seen with as required treatment was less satisfactory than with planned chemotherapy.


Br. J. Cancer (1991), 64, 566-572 © Macmillan Press Ltd., 1991

### A randomised trial of planned versus as required chemotherapy in small cell lung cancer: a Cancer Research Campaign trial

H.M. Earl[1], R.M. Rudd[2], S.G. Spiro[3], C.M. Ash[1], L.E. James[1], C.S. Law[1], J.S. Tobias[1], P.G. Harper[4], D.M. Geddes[3], D. Eraut[5], M.R. Partridge[6] & R.L. Souhami[1]

[1]*Department of Oncology, University College and Middlesex School of Medicine, 91 Riding House Street, London W1P 8BT;* [2]*London Chest Hospital, Bonner Road, London E2 9JX;* [3]*Brompton Hospital, Fulham Road, London SW3 6HB;* [4]*Guy's Hospital, St Thomas's Street, London SE1 9RT;* [5]*Southend Hospital, Westcliff-on-Sea, Essex SS0 0RY;* [6]*Whipps Cross Hospital, London E11 1NR, UK.*

**Summary** In a study of chemotherapy as palliative treatment, 300 patients with untreated limited and extensive stage small cell lung cancer (SCLC), who did not have progressive disease after the first cycle of chemotherapy, were randomised to receive either regular 'planned' chemotherapy or chemotherapy given 'as required' (AR). All patients received the same chemotherapy: cyclophosphamide 1 gm $m^{-2}$ i.v., vincristine 2 mg i.v., and etoposide 120 mg $m^{-2}$ i.v. on day 1, and etoposide 100 mg b.d. orally on days 2 and 3. Planned chemotherapy was given regularly every 3 weeks. AR chemotherapy was given for tumour-related symptoms, or for radiological progression of disease. Both groups of patients were assessed every 3 weeks and a maximum of eight cycles of chemotherapy was given. A detailed quality of life assessment was made using daily diary cards.

The median survival (MS) of patients given AR chemotherapy was not significantly worse than those receiving planned treatment [MS: Planned = 36 weeks (95% C.I. 32–40 weeks), AR = 32 weeks (95% C.I. 28–37 weeks) $P = 0.960$]. In the AR patients the median interval between treatments was 42 days. On average AR patients received half as much chemotherapy as planned patients. AR patients with a treatment-free interval (TFI) of more than 8 weeks between the first and second cycles of chemotherapy survived longer than those in whom this interval was less than 4 weeks; [MS: TFI $>8 = 47$ weeks (95% C.I. 32–53 weeks); TFI $<4 = 24$ weeks (95% C.I. 17–34 weeks) $P = 0.013$]. Contrary to expectation, in the quality of life assessment the AR patients scored themselves as having more severe symptoms than patients receiving planned treatment.

AR chemotherapy is a novel method of attempting to use cytotoxic drugs palliatively, which resulted in less drug treatment for approximately equivalent survival. However the palliative effect seen with as required treatment was less satisfactory than with planned chemotherapy.

Small cell lung cancer (SCLC) is sensitive to both chemotherapy and radiotherapy. Chemotherapy response rates of greater than 80% are obtained in previously untreated patients (Klastersky *et al.*, 1982; Aisner *et al.*, 1986; Feld *et al.*, 1987; Smith *et al.*, 1987; Jackson *et al.*, 1988; Spiro *et al.*, 1989), and chemotherapy prolongs survival in patients with both limited and extensive disease. Unfortunately these high response rates do not result in significant numbers of cures, and the overall 2-year survival is 5.9%, with 3% alive at 7 years (Souhami & Law, 1990). Survival beyond 2 years is largely confined to patients with limited disease and good performance status who constitute only 25% of all cases (Osterlind & Andersen, 1986; Souhami & Law, 1990). Chemotherapy relieves symptoms and increases median survival even in patients who cannot be cured, but these benefits have to be weighed against the toxicity and inconvenience of treatment. In the course of a study designed to assess the optimum duration of chemotherapy (Spiro *et al.*, 1989), a quality of life analysis suggested that symptoms improved if chemotherapy was stopped early (Geddes *et al.*, 1990).

The present study was designed to assess two different philosophies of treatment with cytotoxic drugs. In the first, chemotherapy was used in the conventional manner, given in regular planned cycles every 3 weeks. In the second, chemotherapy was given only when the progression of the disease or the development of symptoms dictated the need for chemotherapy. A pilot study was first undertaken, and the approach was feasible provided that the indications for treating with chemotherapy were made clear. The two most important end points of this study were therefore overall survival and detailed assessment of quality of life. The quality of life assessment was with daily diary cards as described previously (Geddes *et al.*, 1990).

Correspondence: R.L. Souhami.
Supported by a grant from The Cancer Research Campaign.
Received 7 January 1991; and in revised form 14 May 1991.

### Patients and methods

During the period February 1986 and September 1988, 300 patients with SCLC were entered into the study from the participating hospitals. All patients had SCLC diagnosed by histology (from bronchial biopsy, lymph node biopsy, or biopsy, or biopsy of a metastasis), or by cytology either from bronchial brushings at bronchoscopy or from two specimens of sputum. Patients were under 76 years of age at the time of diagnosis, and had no cardiac, renal or neurological disease that would preclude the use of chemotherapy. All those patients with SCLC presenting to the participating centres who were judged to have a survival of more than 3 weeks were entered into the study. There were no patients who refused to be entered. Patients were excluded if they had been treated for another malignant disease within the previous 3 years (except basal cell skin carcinoma), or if they had received previous chemotherapy or radiotherapy for SCLC (apart from those who received emergency radiotherapy for superior vena vacal obstruction, or spinal cord compression).

Patients were staged by chest X-ray, full blood count, and measurement of blood urea, electrolytes, liver function tests, plasma proteins and calcium estimations, isotope bone scan and liver ultrasound. Bone marrow examination was carried out when indicated by an abnormal full blood count. Limited disease (LD) was defined as disease confined to one hemithorax or ipsilateral supraclavicular nodes. Extensive disease (ED) was defined as more widespread local disease, or the presence of metastases. Informed consent was obtained from all patients according to the requirements specified by the individual ethical committees of each of the participating institutions.

Patients were randomised after diagnosis and staging. Randomisation was either to receive planned chemotherapy every 3 weeks, or to receive chemotherapy on an 'as required' (AR) basis. All patients received the first cycle of chemotherapy on entry to the study but patients in either arm who had progressive disease at 3 weeks were taken off the protocol treatment ('off study'). The comparison is therefore between the two treatment policies in those patients with stable or responding disease following the first cycle. Survival data are presented both for all randomised patients and for those on study following the first treatment. In both arms of the study, the chemotherapy was cyclophosphamide 1 gm $m^{-2}$ i.v., vincristine 2 mg i.v., and etoposide 120 mg $m^{-2}$ i.v., on day 1, and etoposide 100 mg b.d. orally on days 2 and 3. Each patient was reviewed every 3 weeks by clinical assessment, chest radiograph, full blood count, urea, electrolytes and liver function tests. A maximum of eight cycles of chemotherapy was given. Protocol chemotherapy was discontinued if there was progressive disease within 3 weeks of previous chemotherapy or if there had been unacceptable chemotherapy side effects. Treatment was given every 3 weeks in the planned group provided that the total white cell count on the day of treatment was equal to or greater than $3.5 \times 10^9 l^{-1}$ and the platelet count was equal to or greater than $100 \times 10^9 l^{-1}$. If not, treatment dosage was reduced according to the following schedule: if the total white cell count was $3-3.5 \times 10^9 l^{-1}$, 75% of the cyclophosphamide and of the intravenous etoposide was given, and if less than $3 \times 10^9$ l, the treatment was not given and the blood count was repeated a week later; if the platelet count was $75-100 \times 10^9 l^{-1}$, cyclophosphamide and etoposide doeses were reduced to 75%; any further decrease in platelet count caused the treatment to be delayed and the blood count was repeated a week later. These dose reductions were carried over to subsequent treatment cycles. After the first course of chemotherapy all patients had a blood count at 7–10 days. If the total white cell count was $< 2 \times 10^9 l^{-1}$ patients were treated prophylactically with Co-trimoxazole two tablets b.d., to avoid the high risk of treatment related mortality at 7–12 days following the first cycle in poor prognosis patients (Moritty *et al.*, 1989).

Patients receiving chemotherapy in the AR arm were assessed every 3 weeks in the same way, but were not treated unless it was 'required'. Treatment decisions were made according to the guidelines in Table I. The same dose modifications were made in the AR arm as in the planned arm, although because treatment was usually given at intervals greater than 3 weeks, chemotherapy was rarely delayed as a result of myelosuppression.

Patients whose tumour progressed within 3 weeks of previous chemotherapy were treated symptomatically including the use of palliative radiotherapy but not further chemotherapy. Patients whose tumours relapsed after chemotherapy had been discontinued were also treated symptomatically. Thoracic radiotherapy and prophylactic cranial irradiation were not given as a part of the treatment protocol.

### Response criteria

Response was assessed clinically, radiologically and biochemically before each chemotherapy cycle. A complete response (CR) was defined as complete radiological clearing of the chest radiograph abnormality seen at diagnosis. All symptoms and signs and biochemical abnormalities indicating metastatic disease should have resolved completely. Bronchoscopic confirmation of complete response was not required. A partial response (PR) was a 50% or greater reduction in tumour area as measured by the sum of two straight lines drawn across the tumour at right angles to each other. Complete and partial responses had to be maintained for at least 3 weeks (the time between two chemotherapy cycles). Stable disease (SD) was either no change in the size of the tumour, or any response that was less than 50%. Progressive disease (PD) was recorded if the tumour increased in size by more than 25%, or the patient developed a new metastasis. Liver relapse was diagnosed with deteriorating liver function tests, and an abnormal isotope or ultrasound scan. If biochemical deterioration in liver function tests occurred as an isolated feature, this was judged to be due to metastatic disease if the abnormality was sustained or increased at the next visit. Bone pain was interpreted as due to metastatic disease if associated with either appropriate X-ray changes or a positive bone scan. Relapse in lymph node or skin lesions was confirmed by cytology or histology only if there was doubt as to their nature. CNS relapse was confirmed by CT brain scan, and carcinomatous meningitis by examination of the CSF. Change in blood urea and electrolytes possibly attributable to ectopic ADP production was not interpreted as relapse if it occurred in insolation.

**Table I** Reasons for treating or not treating patients on 'as required' chemotherapy

| *Reasons for treating with chemotherapy* | *Reasons for not treating with chemotherapy* |
|---|---|
| Responding but symptomatic | Responding and asymptomatic |
| SD and symptomatic | SD and asymptomatic |
| PD but asymptomatic | PD despite treatment |
| PD and symptomatic | |

PD = Progressive disease. SD = Stable disease.

### Statistical methods

In this study we were comparing a standard treatment with a more conservative treatment. The hypothesis being tested was that the two treatments would be similar in terms of survival, but that the quality of life might be better for the AR patients. The objective was the identification of the less toxic treatment, provided it was not worse than the standard treatment in terms of survival. The acceptable difference in survival was agreed by the investigators at the start of the study. With an expected 30% survival at 1 year, we wished to be able to detect a > 15% difference in survival at 1 year. Using the method of Makuch and Simon (1978) the total number of patients required ($\alpha = 0.10$, $\beta = 0.20$ and $\sigma = 0.15$) was 230 (115 in each arm). Alternatively, statistical methods using a one-tailed test (Freedman, 1982) indicated a total of 242 patients would be required when $\alpha = 5\%$, $1\text{-}\beta = 80\%$, with an expected 30% 1 year survival, and the ability to detect a 15% difference.

Patients were randomised centrally by the trial coordinator using the sealed envelope method. The randomisations were stratified according to treatment centre. Curves of survival and treatment free interval were constructed according to the method of Kaplan and Meier (1958) and statistical significance estimated by the log-rank test (Peto *et al.*, 1977).

### Quality of life assessment

A cohort of 62 patients taking part in the multi-institutional randomised study also participated in the quality of life assessment. The patients were treated in a single institution (London Chest Hospital). They were under the care of a single medical team and were judged to be capable of complying with the diary card assessment. After diagnosis patients were asked to participate in the study and gave informed consent. All patients were aware of their diagnosis. The nature of the trial was explained by one of two doctors. The intention behind 'planned' and 'as required' chemotherapy was explained in the same way to each patient. Patients allocated to as required chemotherapy were told that the progress of the disease would be closely monitored and chemotherapy used as and when necessary. No patient refused to take part.

The quality of life measure used was a diary card to be completed daily by the patient (Figure 1). This card has been compared by our group in a previous study (Geddes *et al.*, 1990) with the EORTC questionnaire, the Spitzer quality of life index and the HAD scale and is a modification of the card developed by the UK Medical Research Council (Bleehen *et al.*, 1989). The diary card was shown to be sensitive to short term day to day changes in mood, and to

PLEASE ANSWER THE FOLLOWING QUESTIONS.
WRITE DOWN THE NUMBER OF YOUR ANSWER
IN THE APPROPRIATE BOX OPPOSITE THIS PAGE.

WEEK 1

| | Mon | Tues | Wed | Thur | Fri | Sat | Sun | |
|---|---|---|---|---|---|---|---|---|
| DID YOU FEEL SICK TODAY?<br>1 Not at all 2 Occasionally<br>3 A lot 4 All the time | | | | | | | | |
| DID YOU VOMIT TODAY?<br>1 Not at all 2 Once<br>3 Twice 4 More than twice | | | | | | | | |
| HOW GOOD HAS YOUR APPETITE BEEN TODAY?<br>1 Good 2 Fair<br>3 Poor 4 Bad | | | | | | | | |
| HOW MUCH PAIN HAVE YOU HAD TODAY?<br>1 None 2 A little<br>3 Quite a lot 4 A lot | | | | | | | | |
| HOW DID YOU SLEEP LAST NIGHT?<br>1 Very well 2 Quite well<br>3 Badly 4 Not at all | | | | | | | | |
| HOW HAPPY HAVE YOU BEEN TODAY?<br>1 Happy 2 Fairly happy<br>3 Unhappy 4 Very unhappy | | | | | | | | |
| HOW ARE YOU FEELING GENERALLY?<br>1 Well 2 Fair<br>3 Poor 4 Very poor | | | | | | | | |
| WHAT DID YOU DO TODAY?<br>1 Stayed in bed 2 Got up – did nothing<br>3 Light work/House work 4 Fully active | | | | | | | | |

**Figure 1** The daily diary card.

symptoms related to both the chemotherapy cycles and the disease (Geddes *et al.*, 1990). These findings have been confirmed by Fayers *et al.* (in press). The nature of the diary card was explained to the patient by a single research nurse at the time of their first treatment. The patients were shown how to complete it at the end of each day. The research nurse checked the card at each visit and remained in contact with the patient throughout the course of his or her illness. The cards covered a 4 week period (to allow for treatment delays) and were collected at each hospital or clinic visit, when a new card was supplied. Eight questions were asked and the patient responded by choosing the most appropriate answer on a four point categorical scale according to the severity of their symptoms. The questions were designed to cover three areas: symptoms related mainly to treatment sickness, vomiting, appetite; symptom related to disease – pain; and a more general assessment – mood, sleep, activity and general well being. Patients were encouraged to complete the diary cards for as long as possible to enable a comparison of the two arms when those receiving planned chemotherapy had completed eight courses and those on the AR arm were still eligible for treatment. Results are expressed graphically as the proportion of the total weekly scores for all patients which were scored as greater than grade 1. We have previously shown (Geddes *et al.*, 1990) that this trend is identical (but the proportion lower) if the cut-off point is taken as greater than 2 or 3. For comparison between results in the diary card the Mann-Whitney non-parametric test was used.

### Results

Three hundred patients entered the study between February 1986 and September 1988, 155 to the planned arm and 145 to the AR arm. After the first cycle of chemotherapy when the patients were reassessed at 3 weeks, 23 patients (15%) from the planned arm, and 25 patients (17%) from the AR arm did not receive further chemotherapy on study and Table II states the reasons. One hundred and thirty two patients in the planned and 120 patients in the AR arm went on to receive further treatment. Table III shows the patient characteristics in the two arms of the study, both including and excluding the patients who progressed after the first cycle. Both groups were well matched for age, sex, performance status (PS) and biochemical features. All 132 patients in the planned arm of the study have completed their chemotherapy, whereas there are seven patients on the AR arm who are still eligible to receive more chemotherapy at the time of this analysis. At the present time these patients remain in either complete or partial remission and are

**Table II** Reasons for treatment being discontinued after course one

| | *Planned* | *As required* |
|---|---|---|
| Progressive disease by Day 21 | 10 | 12 |
| Death before Day 21 | 6 | 10 |
| Patient withdrawal before Day 21 | 2 | 2 |
| Diagnosis incorrect | 3 | – |
| Other | *2* | *1* |
| | 23 | 25 |

**Table III** Patient characteristics. Responding patients are patients who did not have progressive disease after the first cycle

| | *All patients* | | *Responding patients* | |
|---|---|---|---|---|
| | *Planned (155)* | *As required (145)* | *Planned (132)* | *As required (120)* |
| Age | | | | |
| Range | 39–75 | 43–75 | 39–75 | 43–75 |
| Median | 65 | 66 | 64 | 65 |
| Stage | | | | |
| L | 46 (29.7 %) | 49 (33.8%) | 41 (31.1%) | 39 (32.5%) |
| E | 109 (70.3%) | 96 (66.2%) | 91 (68.9%) | 81 (67.5%) |
| Sex | | | | |
| M | 119 (76.8%) | 105 (72.4%) | 102 (77.3%) | 88 (73.3%) |
| F | 36 (23.2%) | 40 (27.6%) | 30 (22.7%) | 32 (26.7%) |
| PS | | | | |
| 0 | 43 (27.7%) | 44 (30.3%) | 38 (28.8%) | 42 (35%) |
| 1 | 64 (41.3%) | 50 (34.5%) | 58 (43.9%) | 44 (36.7%) |
| 2 | 25 (16.2%) | 25 (17.2%) | 20 (15.2%) | 20 (16.7%) |
| 3 | 23 (14.8%) | 26 (18%) | 15 (11.4%) | 14 (11.6%) |
| Plasma Albumin[a] | 89.66 | 89.41 | 89.66 | 91.11 |
| Plasma Na[a] | 97.84 | 97.84 | 97.84 | 97.86 |
| Alkaline P'ase[a] | 97.06 | 105.88 | 97.14 | 104.00 |

[a]Values are median value of all patients expressed as % of the mean of the normal range of each participating institution. L = Limited disease. E = Extensive disease.

asymptomatic from their disease. Four patients on the planned arm and seven patients on the AR arm withdrew from treatment. Only one patient was lost to follow-up in the study (AR arm).

The number of courses of chemotherapy received by the patients is shown in Figure 2. 56.8% of patients on the planned arm have received all eight courses of chemotherapy, compared with 12.4% of patients on the AR arm. The median number of courses received by patients on the planned arm of the study was eight, compared with four for patients on the AR arm. Dose reductions were infrequent. At the 8th cycle the mean percentage intravenous dose was 91.7% in the planned arm and 95.8% in the as required. The overall per cent dose per cycle was 91.7% in the planned and 96.6% in the as required treatment. In the planned arm 12.6% of cycles were delayed by 1 week.

Figure 3 shows the overall survival for all 300 patients randomised. There was no significant difference in survival between all patients randomised to receive planned or AR chemotherapy, with median survivals of 35 weeks (95% C.I. 30–39 weeks) and 29 weeks (95% C.I. 25–33 weeks) respectively ($P = 0.464$). There was no difference in overall survival between the 252 patients who, with stable or responding disease after the first cycle, received either planned or AR chemotherapy, with median survivals of 36 (95% C.I. 32–40 weeks) and 32 (95% C.I. 28–37 weeks) weeks respectively (Figure 4, $P = 0.960$). There was no difference in survival between the two groups in patients with stable or responding disease in the limited or extensive disease category (Figure 5a and b).

**Figure 2** The number of courses received by all patients entered onto study. ▨ = AR chemotherapy, ■ = Planned chemotherapy.

The median interval between treatments in the AR arm was 42 days. Figure 6 and the legend shows the method of calculation and analysis of the treatment-free intervals between each course. There are only minor changes in median treatment free interval from course 1 through 8. It is of note that approximately 10% of patients had treatment-free intervals of 3 months or longer.

Figure 7 demonstrates overall survival by duration of first treatment interval. Patients with a first treatment-free interval of <4 weeks have a median survival of 24 weeks (95% C.I. 17–34 weeks); patients with an interval of 4–8 weeks have a median survival of 33 weeks (95% C.I. 31–37 weeks); and those with a first interval of > 8 weeks have a median survival of 47 weeks (95% C.I. 32–53 weeks, trend test $P = 0.013$).

### Quality of life assessment

The characteristics of the patients studied in the quality of life assessment and in the whole trial are compared in Table IV. The study patients are representative of the trial as a whole and the two arms are evenly matched. Table V shows the number of patients studied during the period of chemotherapy. During the period of observations patients continued to fill in diary cards after relapse until they were too ill to comply when they withdrew from the assessment. This is the explanation of the diminution in numbers with time. Table VI shows the numbers of patients treated for each chemotherapy cycle. As in the whole study, patients on the AR arm received approximately half the amount of chemotherapy given to the patients on the planned programme (106 cycles *vs* 196) before relapse within 3 weeks of the last cycle. Patient compliance in return of completed cards was excellent, with 438 out of a possible 506 (87%) cards being returned (88% planned and 85% AR). Figure 8 shows the proportion of responses indicating nausea, vomiting, depression of appetite and pain greater than grade 1 in both study groups. The peaks of nausea related to the chemotherapy cycles in the planned arm are clearly demonstrated. They are less apparent in the AR arm since chemotherapy occurred at differing time intervals. In the planned arm nausea diminished at week 22 when chemotherapy was discontinued. Nausea continues longer in the

**Figure 3** Overall survival for all randomised patients according to randomisation. **a**, AR chemotherapy ($n = 145$, MS = 29 weeks, 95% C.I. 25–33 weeks; observed deaths 134, expected deaths 128). **b**, Planned chemotherapy ($n = 155$, MS = 35 weeks, 95% C.I. 30–39 weeks; observed deaths 141, expected deaths 147) $P = 0.464$.

**Figure 4** Overall survival for all patients with stable or responding disease after the 1st cycle according to randomisation. **A**, AR chemotherapy ($n = 120$, MS = 32 weeks, 95% C.I. 28–37 weeks; observed deaths 112, expected deaths 112.) **B**, Planned chemotherapy ($n = 132$, MS = 36 weeks, 95% C.I. 32–40 weeks; observed deaths 124, expected deaths 124), $P = 0.960$.

**Figure 5** a: Overall survival for limited disease patients with stable or responding disease after the first cycle according to randomisation. A, AR chemotherapy ($n$ = 39, MS = 47 weeks, 95% C.I. 35–61 weeks; observed deaths 36, expected deaths 39). B, Planned chemotherapy ($n$ = 41, MS = 43 weeks, 95% C.I. 30–49 weeks; observed deaths 36, expected deaths 33); $P$ = 0.495. b: Overall survival for extensive disease patients with stable or responding disease after the first cycle according to randomisation. A, AR chemotherapy ($n$ = 81, MS = 28 weeks, 95% C.I. 23–32 weeks; observed deaths 76, expected deaths 71). B, Planned chemotherapy ($n$ = 91, MS = 35 weeks, 95% C.I. 30–39 weeks; observed deaths 88, expected deaths 93), $P$ = 0.464.

**Figure 6** Treatment-free interval curves for as required chemotherapy patients only. The individual curves are not identified since there is no difference between them. The curves are constructed on the same principle as survival curves. A fall in the curve indicates that a patient has received treatment, and the points indicate that a patient has been treatment-free for that length of time since their last treatment. The treatment-free intervals pertain to those chemotherapy cycles before progression through treatment occurs. The median treatment-free interval (MTFI) between each course is as follows: Course interval 1–2 ($n$ = 147, MTFI = 43 days); Course 2–3 ($n$ = 121, MTFI = 42 days); Course 3–4 ($n$ = 99; MTFI = 43 days); Course 4–5 ($n$ = 72, MTFI = 43 days; Course 5–6 ($n$ = 55, MTFI = 41 days); Course 6–7 ($n$ = 36, MTFI = 42 days); Course 7–8 ($n$ = 20, MTFI = 41 days). Log Rank test $P$ = 0.628. Trend Test $P$ = 0.197.

**Figure 7** Overall survival by duration of first treatment-free interval, for AR chemotherapy patients only. a, first interval <4 weeks ($n$ = 51, MS = 24 weeks, 95% C.I. 17–34 weeks). b, First interval 4–8 weeks ($n$ = 53, MS = 33 weeks, 95% C.I. 31–37 weeks). c, First interval >8 weeks ($n$ = 43, MS = 47 weeks, 95% C.I. 32–53 weeks). Trend Test $P$ = 0.013.

**Table IV** Characteristics of the patients in the quality of life study compared with all randomised patients with stable or responding disease after the first cycle

| | *Planned* | | *As required* | |
|---|---|---|---|---|
| *PS (%)* | *Study group* | *Entire study* | *Study group* | *Entire study* |
| 0 | 33.3 | 29.0 | 33.3 | 35.0 |
| 1 | 53.3 | 44.3 | 43.3 | 36.7 |
| 2 | 10.0 | 15.3 | 13.3 | 16.7 |
| 3 | 3.3 | 11.4 | 10.0 | 11.6 |
| LD (%) | 24.1 | 31.1 | 33.3 | 32.5 |
| ED (%) | 75.9 | 68.9 | 66.7 | 67.5 |

PS = Performance status. LD = Localised disease. ED = Extensive disease.

**Table V** Numbers of patients returning cards during study period

| *Week* | *1* | *4* | *8* | *12* | *16* | *20* | *24* | *28* |
|---|---|---|---|---|---|---|---|---|
| Planned | 32 | 29 | 27 | 26 | 23 | 21 | 17 | 17 |
| As required | 30 | 27 | 26 | 24 | 22 | 20 | 18 | 13 |

AR arm because chemotherapy continues longer. The important observation is that there was no general reduction in the level of nausea in the AR arm. Similar results (but for a smaller proportion of patients) are obtained if grade 2 is used as the cut-off point. These results are confirmatory of earlier reports of the use of cards (Geddes et al., 1990). There are more high scores for vomiting in the planned arm who are receiving more chemotherapy cycles than AR patients, but appetite shows a steady deterioration in the AR arm. Symptoms of pain were also more frequent in the AR group.

Figure 9 shows the results for sleep, mood, activity and general well being. Disturbances of mood and sleep are more frequent in the AR group. More of these patients also show a deterioration in what we have described as 'general well being'. Activity scores were slightly worse in the planned group, possibly related to the greater number of hospital admissions (note that activity is scored inversely, 100% being all patients fully active).

### Discussion

This study in patients with small cell lung cancer was designed to answer a novel question in the use of cancer chemotherapy. Does chemotherapy treatment given on an 'as required' basis result in equivalent survival with a better quality of life? Because the prognosis for SCLC remains very

**Table VI** Numbers of patients treated at each chemotherapy cycle

| *Chemotherapy cycle* | *1* | *2* | *3* | *4* | *5* | *6* | *7* | *8* |
|---|---|---|---|---|---|---|---|---|
| Planned | 32 | 29 | 27 | 26 | 23 | 21 | 21 | 17 |
| (3 weekly) | 30 | 23 | 21 | 15 | 9 | 6 | 2 | 1 |
| As required | | | | | | | | |
| Week given: median (range) | | 7 (4–23) | 13 (7–29) | 19 (10–37) | 25 (14–48) | 27 (17–39) | | |

**Figure 8** The percentage of weekly scores reporting symptoms of grade 1 or more for nausea, vomiting, appetite, pain. There was no significant difference in scores for vomiting ($P = 0.30$) but nausea ($P = <0.001$), appetite ($P = <0.001$), pain ($P = <0.001$) were more adversely affected in the AR group. ■ = AR group. O = Planned group.

**Figure 9** The percentage of weekly scores reporting symptoms of grade 1 or more for sleep, mood, general well being and activity. Mood ($P = 0.001$, sleep ($P = <0.001$) and general well being ($P = <0.001$) were adversely affected in the AR group. Activity was worse ($P = <0.05$) in the planned group (note that activity is scored in the opposite direction to the other symptoms – a high score indicating more activity). ■ = AR group. O = Planned group.

poor, questions concerning the optimum use of chemotherapy for the aleviation of symptoms are of considerable importance. The concept of 'as required' chemotherapy can only ethically be adopted when the aims of the study are not curative but palliative, since chemotherapy given 'as required' is unlikely to be curative.

This study did not detect a significant worsening of survival as the result of the use of chemotherapy on an AR basis. AR chemotherapy is therefore a feasible approach to treatment in SCLC. Patients randomised to the planned arm received on average twice as much chemotherapy as patients treated on an AR basis. The treatment-free intervals in those receiving AR chemotherapy remained relatively constant throughout the course of treatment with a median of 6 weeks. The low overall median survivals for both limited and extensive disease in this unselected group of patients is in keeping with data from other large centres (Osterlind & Andersen, 1986).

It is of interest that the regrowth interval of the tumour appeared to have an association with prognosis. The longer regrowth interval may be due either to a greater reduction in tumour mass as a result of chemotherapy, or an intrinsically slower growth rate. The fact that many patients with only a partial response on X-ray showed a very slow regrowth of the tumour suggests that the distribution of growth rates in SCLC may be wider than is generaly assumed. Patients whose tumour responded well to treatment and who had long treatment-free intervals, had a better survival.

To assess quality of life we used the daily diary card which we have shown gives detailed information on short-term variations in symptoms which may not be detected by more general questionnaires (Geddes *et al.*, 1990). Compliance, which is often a problem with self administered assessments (Bleehen *et al.*, 1989), was good, probably due to the fact that the study was carried out in a single centre with a research nurse assigned solely for this purpose. Patients in both arms of the study continued to complete cards for as long as possible. In the previous study (Geddes *et al.*, 1990) a decision was made to stop the assessment once progression occurred. This was not appropriate when comparing planned and as required chemotherapy, since in those randomised to AR chemotherapy treatment was given only on disease progression or persistence of symptoms. However many patients die in the first few months of treatment and only 30 of an initial 62 patients were completing cards by week 28. Nevertheless almost 450 cards were collected, each containing at least 160 scores and compliance in each group was very similar.

The results confirm our previous report and that of Fayers *et al.* (in press) that the diary card was sensitive enough to detect the changing symptoms during treatment cycles and also to show clear differences between the two treatment policies. The diary card produces large amounts of repeated data. In displaying and analysing the two groups the percentage of scores above (or below) a given level (1, 2, 3 or 4) was calculated for each symptom category, for all patients, over a period of 1 week. Although in theory it might be possible to achieve the same results by collecting the data less frequently, it was felt that the regularity of a daily recording was an important factor in compliance. Following the suggestions of Fayers and Jones (1983), who argue against over-sophisticated analysis of the data, both the raw data and the weekly proportions were compared using the Mann-Whitney non-parametric test which confirmed the differences already evident in the graphical display shown in the figures. The results are shown for scores greater than one. An identical trend was observed if scores greater than two were taken. Too few patients had scores greater than three or four for the analysis to have value.

Giving chemotherapy on an AR basis proved to be a successful way of reducing the number of treatments that patients receive. The median treatment free interval was 6 weeks, resulting in AR patients receiving approximately half the number of courses as the planned patients. This reduction in treatments did not lead to a significant survival difference and resulted in one of the main side effects of chemotherapy, that of vomiting, being somewhat reduced. However in all the other diary card measures excepting activity more AR patients reported adverse symptoms.

Two possible explanations might account for the worse quality of life in the AR group. Firstly it is possible that patients felt that this treatment was a philosophy of failure, and were distressed because they felt that they could not be cured. This might have been reinforced each time chemotherapy was given since at that time the disease might be perceived as advancing. However there are arguments against this interpretation. One team of two doctors explained the study to patients who were then closely followed by the research nurse. There were no adverse comments about the trial reported by AR patients and neither the nurse nor the doctors were aware of the trend towards worse symptoms. In fact the treatment was very popular with the medical staff and patients are likely to have perceived this. The deterioration is therefore more likely to be due to the physical effects of cancer which are alleviated by chemotherapy even if at the expense of some toxicity. If this interpretation is correct the study shows that, for most patients, regular chemotherapy is an effective and useful palliative treatment and that while patients dislike the side effects of treatment, failure to control the effects of the cancer diminishes quality of life to a greater degree.

Similar conclusions have been reached in the treatment of metastatic breast cancer. Coates *et al.* (1987) conducted a trial comparing planned, regular, chemotherapy administered until progression of disease, with intermittent chemotherapy, whereby treatment was stopped after 3 cycles and then repeated for three more cycles only when there was evidence of disease progression. Intermittent therapy in this group of patients with metastatic breast cancer resulted in a significantly worse response, a significantly shorter time to disease progression, a trend towards shorter survival and was associated with a worse quality of life than in patients treated with regular, planned course of chemotherapy until disease progression. It would therefore appear that chemotherapy not only prolongs life but also improves its quality, an inference also drawn by Tannock (1987) in reviewing the study of Coates and co-workers (1987). This is an important conclusion for all those involved in the treatment of patients with SCLC and may also have relevance in the palliative treatment of patients with metastatic disease from other tumours that are responsive to chemotherapy. Finally, this study illustrates the value of objective measurement when a question of palliation is being asked and shows that the perception of doctors and nurses may not accord with what the patient experiences.

This study was supported by the Cancer Research Campaign. The computing facilities were made available by the Imperial Cancer Research Fund. Walter Gregory gave statistical advice and Mrs M. Cohen typed the manuscript with great care. We would like to thank the following for their referral or treatment of patients on this study: Dr M. Apps, Dr R. Ashford, Dr L.R. Bagg, Dr R.A. Banks, Dr N. Barnes, Dr D. Barrett, Dr K.M. Citron, Dr M. Cochrane, Dr P. Cole, Dr J.V. Collins, Dr C. Coulter, Dr A.G. Davison, Dr N. Eiser, Dr D.W. Empey, Dr R.W. Fowler, Dr D. Gamble, Dr J.R. Govan, Dr M. Green, Dr M. Henk, Dr M.R. Hetzel, Dr M.E. Hodson, Dr D. Hughes, Dr N. Johnson, Dr W.P. Kennedy, Dr R.K. Knight, Dr V. Levison, Dr W.A.C. McAllister, Dr O. McCarthy, Dr M.W. McNicol, Dr J. Maher, Dr B.S. Mantell, Dr J. Meadway, Dr J. Milledge, Dr D. Mitchell, Dr A. Newman-Taylor, Dr E.L. Offerman, Dr W.R. Pratt, Dr J. Rees, Dr J. Riordan, Dr A. Rostrom, Dr M. Smith, Dr P. Studdy, Dr D. Tong, Dr C. Trask, Dr J. Utting, Dr S.G. Vaidya, Dr J. Waller, Dr J. Warren, Dr J. Wedzicha, Dr J. Whittle, Dr R. Wilson, Dr J. Winter.

### References

AISNER, J., WHITEACRE, M., ABRAMS, J. & PROPERT, K. (1986). Doxorubicin, cyclophosphamide, etoposide and platinum, doxorubicin, cyclophosphamide and etoposide for small cell carcinoma of the lung. *Seminiars in Oncol.*, **13** (Suppl 3), 54.

BLEEHEN, N.M., FAYERS, P.M., GIRLING, D.J. & STEPHENS, R.J. (1989). Survival, adverse reactions and quality of life during combination chemotherapy compared with selective treatment for small cell lung cancer. *Resp. Med.*, **83**, 51.

COATES, A., GEBSKI, V., BISHOP, J.E. & 12 others (1987). Improving the quality of life during chemotherapy for advanced breast cancer. A comparison of intermittent and continuous treatment strategies. *New Engl J. Med.*, **317**, 1490.

FAYERS, P.M. & JONES, D.R. (1983). Measuring and analysing quality of life in cancer trials: a review. *Stats. Med.*, **2**, 429.

FAYERS, P.M., BLEEHEN, N.W., GIRLING, D.J. & STEPHENS, R.J. (in press). Assessment of quality of life in small cell lung cancer using a daily diary card developed by the Medical Research Council Lung Cancer Working Party.

FELD, R., EVANS, W.K., COY, P. & 6 others (1987). Canadian multicenter randomised trial comparing sequential and alternating non-cross-resistant chemotherapy combinations in patients with limited small cell carcinoma of the lung. *J. Clin. Oncol.*, **5**, 1401.

FREEDMAN, L. (1982). Tables of the number of patients required in clinical trials using the log rank test. *Stats. Med.*, **1**, 121.

GEDDRES, D.M., DONES, L., HILL, E. & 5 others (1990). Quality of life during chemotherapy for small cell lung cancer: use and validation of a daily diary card in a randomised trial. *Eur. J. Cancer Clin. Oncol.*, **26**, 484.

JACKSON, D.V., CASE, L.D., ZEKAN, P.J. & 13 others (1988). Improvement of long-term survival in extensive small cell lung cancer. *J. Clin. Oncol.*, **6**, 1161.

KAPLAN, E.L. & MEIER, P. (1958). Non-parametric estimations from incomplete observations. *J. Am. Stat. Assoc.*, **53**, 457.

KLASTERKSY, J., NICAISE, C., LONGVAL, E., STYCKMANS, P. & THE EORTC LUNG CANCER WORKING PARTY. (1982). Cisplatin, Adriamycin, and etoposide (CAV) for remission induction of small cell bronchogenic carcinoma. Evaluation of efficacy and toxicity and pilot study of a 'late intensification' with autologous bone marrow rescue. *Cancer*, **50**, 652.

MAKUCH, R. & SIMON, R. (1978). Sample size requirements for evaluating a conservative therapy. *Cancer Treat. Rep.*, **62**, 1037.

MORITTU, L., EARL, H.M., SOUHAMI, R.L. & 5 others (1989). Patients at risk of chemotherapy associated toxicity in small cell lung cancer. *Br. J. Cancer*, **59**, 801.

OSTERLIND, K. & ANDERSEN, P.K. (1986). A model for survival in small cell lung cancer. A study of prognostic factors in 874 patients treated with chemotherapy with or without irradiation. *Cancer Res.*, **46**, 4189.

PETO, R., PIKE, M.C., ARMITAGE, P. & 7 others (1977). Design and analysis of randomised clinical trials requiring prolonged observation of each patient. *Br. J. Cancer*, **35**, 1.

SMITH, I.E., EVANS, B.D., GORE, M.E. & 4 others (1987). Carboplatin (paraplatin; JM8) and etoposide (VP-16) as first-line combination chemotherapy for small cell lung cancer. *J. Clin. Oncol.*, **5**, 185.

SOUHAMI, R.L. & LAW, K.S. (1990). Longevity in small cell lung cancer. A report to the lung cancer subcommittee of the United Kingdom Coordinating Committee for Cancer Research. *Br. J. Cancer*, **61**, 584.

SPIRO, S.G., SOUHAMI, R.L., GEDDES, D.M. & 6 others (1989). Duration of chemotherapy in small cell lung cancer. A Cancer Research Campaign Trial. *Br. J. Cancer*, **59**, 578.

TANNOCK, I.F. (1987). Treating the patient not just the cancer. *New Engl. J. Med.*, **317**, 1534.